# Multimodal Management of Calvarial Osteosarcoma with en Bloc Craniectomy and Adjuvant Imatinib Therapy in a Dog

**DOI:** 10.3390/vetsci13090900

**Published:** 2026-09-02

**Authors:** Yijae Kim, Wookhun Chung, Jaewoong Han, Byung-Joon Seung, Jungwhan Chon, Kihoon Kim

**Affiliations:** 1 Seoul High Animal Medical Center, Paju 10865, Republic of Korea; y2k6565@naver.com; 2 Nowon N Animal Medical Center, Seoul 01704, Republic of Korea; 3 Department of Pathology, College of Medicine, Kangwon National University, Chuncheon 24341, Republic of Korea; bjseung@kangwon.ac.kr; 4 Department of Food Science and Biotechnology, Kangwon National University, Chuncheon 24341, Republic of Korea; 1000cda@kangwon.ac.kr; 5 Department of Surgery, College of Veterinary Medicine, Kangwon National University, Chuncheon 24341, Republic of Korea

**Keywords:** osteosarcoma, craniectomy, dog, imatinib

## Abstract

An osteosarcoma (OSA) is a malignant mesenchymal tumor characterized by osteoid production by neoplastic osteoblasts. Particularly, calvarial OSA is an uncommon form of canine OSA. A 10-year-old male Pomeranian dog presented with a palpable calvarial mass. Computed tomography (CT) demonstrated a well-defined osseous mass arising from the left parietal bone without intracranial invasion or regional metastasis. An en bloc craniectomy including the affected calvarial bone was performed, and histopathological examination confirmed the diagnosis of OSA. Based on previous reports suggesting that PDGFR-mediated signaling may represent a potential therapeutic target in canine OSA, oral imatinib was planned to be administered for 1 year as postoperative adjuvant therapy. Seven months after surgery, follow-up CT showed no evidence of local recurrence or systemic metastatic disease. Ten months after surgery, the dog was re-evaluated for continued anticancer therapy, with no evidence of local recurrence on physical examination. This case suggests that satisfactory local tumor control can be achieved following surgical excision despite anatomically limited surgical margins. In addition, it provides clinical information regarding postoperative imatinib administration and CT follow-up for canine calvarial OSA. To the best of the authors’ knowledge, this is the first report describing adjuvant imatinib therapy following surgical treatment of canine calvarial OSA. However, further studies are warranted to determine the long-term efficacy of this treatment approach.

## Simple Summary

An osteosarcoma (OSA) is the most common primary bone tumor in dogs, but tumors arising from the skull are uncommon and can be difficult to treat because complete removal with adequate margins is often limited by anatomical constraints. This report describes a dog with a calvarial OSA that was evaluated using computed tomography before surgery to determine the exact extent of the tumor. Based on these findings, the lesion, including the mass and grossly normal surrounding bone, was removed and the defect was reconstructed with surgical mesh. After surgery, the dog received oral imatinib, a targeted anticancer drug, for one year as an off-label adjunctive treatment because previous studies have suggested that PDGFR-mediated signaling may represent a potential therapeutic target in canine OSA. Follow-up computed tomography performed seven months after surgery showed no evidence of local tumor recurrence or distant metastasis. At 10 months postoperatively, no local recurrence was evident on physical examination. This case provides clinical information regarding multimodal management of calvarial OSA, including en bloc surgical resection, postoperative adjunctive imatinib administration, and CT-based follow-up.

## 1. Introduction

Osteosarcoma (OSA) is the most common malignant primary bone tumor characterized by the production of osteoid and/or immature bone by neoplastic osteoblasts [1,2]. Approximately 20–25% of canine skeletal OSA arises from the axial skeleton, and OSA of the skull, including the maxilla, mandible, and calvarium, is a relatively uncommon tumor, accounting for 12–13% of all OSA cases [1]. In a retrospective study of 183 dogs, calvarial tumors accounted for only 43 cases (23.5%), whereas maxillary and mandibular tumors accounted for 80 cases (43.7%) and 60 cases (32.8%), respectively [3].

OSA arising from the maxilla, mandible, and calvarium is characterized by locally aggressive behavior, and complete surgical excision remains one of the most important prognostic factors [3]. Although the role of adjuvant therapies, including chemotherapy and radiation therapy, remains poorly defined for OSA of the maxilla, mandible, and calvarium, adjuvant chemotherapy may be considered because of the risk of metastatic disease [3]. According to the veterinary literature, several antitumor agents, including doxorubicin, carboplatin, losartan, and toceranib, have been used as adjuvant treatments for canine OSA [2,4]. Recently, a favorable short-term response to high-dose losartan-based combination therapy has been reported in canine metastatic OSA [2]. In addition, PDGFR-mediated signaling has been investigated as a potential therapeutic target in canine OSA. Previous studies have demonstrated PDGFR-α and PDGFR-β expression in canine OSA tissues and cell lines, suggesting a biological rationale for considering PDGFR-targeted therapy [5]. Imatinib is a tyrosine kinase inhibitor that targets several kinases, including PDGFR-α, PDGFR-β, KIT, and ABL [6,7]. However, the clinical efficacy of imatinib in canine OSA has not been established.

Although OSA of the maxilla, mandible, and calvarium has been described in dogs, calvarial OSA remains uncommon, and information regarding postoperative CT-based surveillance, reconstructive approaches after craniectomy, and targeted adjuvant therapy is limited. Further documentation of the clinical course and postoperative monitoring in individual cases may help improve understanding of this uncommon tumor location. Therefore, the aim of this report was to describe the diagnostic imaging findings, surgical management, calvarial defect reinforcement, postoperative CT monitoring, and adjunctive imatinib therapy in a dog with calvarial OSA.

## 2. Case Description

A 10-year-old male Pomeranian dog weighing 4.56 kg was referred for diagnosis and treatment of a mass in the calvarial region. Physical examination was unremarkable except for a palpable mass over the cranial region. Thoracic radiography revealed no significant findings. Complete blood count and blood chemistry analyses revealed no remarkable change except for mildly increased alkaline phosphatase activity of 157 U/L [Reference range (RR): 20–106 U/L] and total cholesterol concentration of 404 mg/dL (RR: 93–340 mg/dL).

**Figure 1:**
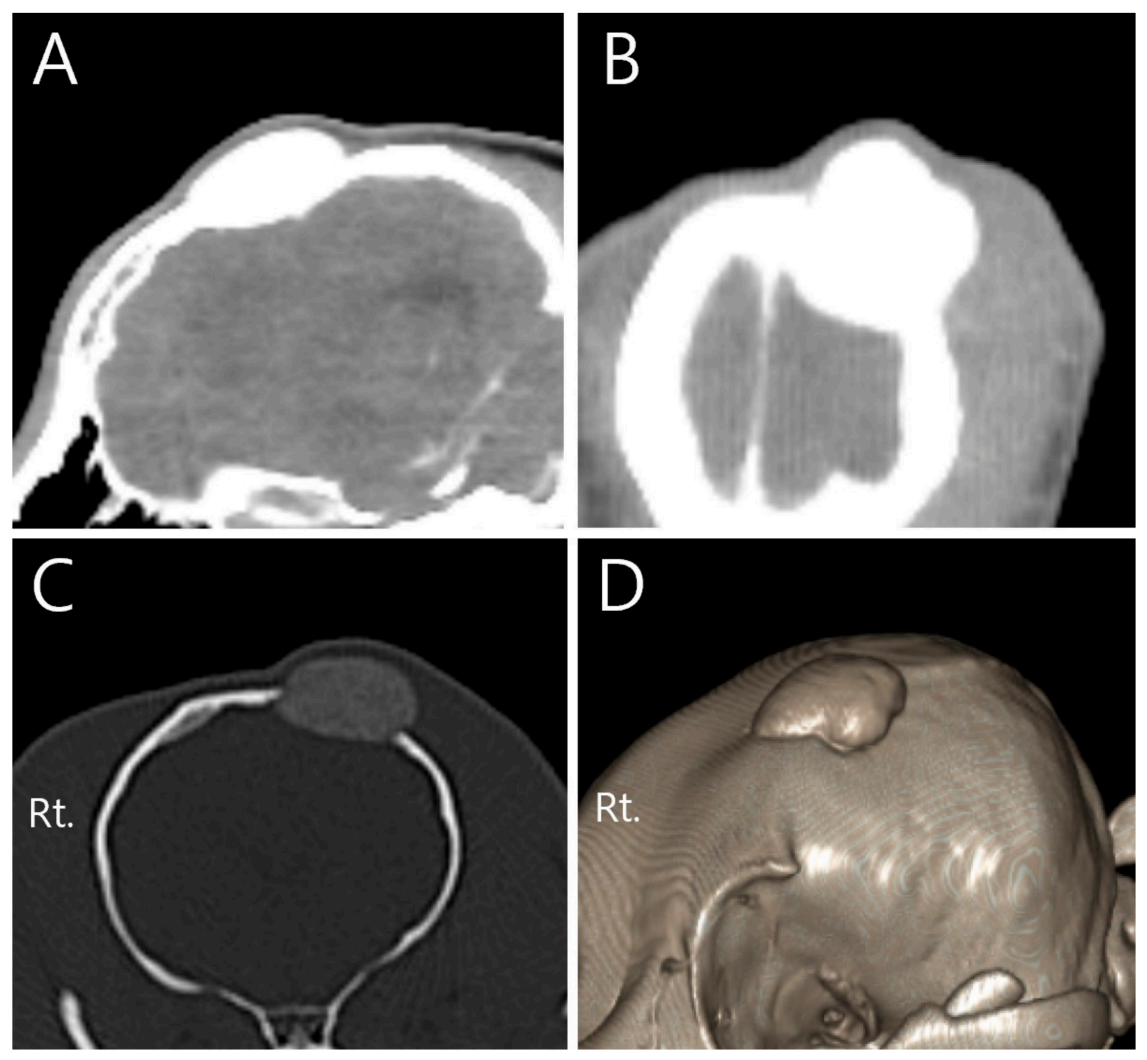
CT images showing calvarial mass. (**A**) Sagittal soft tissue window image demonstrating a well-defined, mild contrast-enhancing, dome-shaped mass arising from the left parietal calvarium. (**B**) Dorsal soft tissue window image showing the rounded extra-cranial mass without apparent invasion of the adjacent soft tissues. (**C**) Transverse bone window image demonstrating an expansile calvarial lesion with thinning of the cortical bone and focal loss of the inner table of the caudal aspect of the skull. No distinct periosteal reaction was identified. (**D**) Three-dimensional volume-rendered reconstruction image showing the exophytic osseous mass originating from the left parietal bone.

Contrast-enhanced CT (Aquilion lightning 160, Canon Medical Systems, Otawara, Japan) demonstrated a well-defined, dome-shaped osseous mass arising from the left parietal calvarium, measuring 18.3 × 16.6 × 9.4 mm (Figure 1A). Whole-body CT examination revealed no evidence of metastatic disease or additional primary neoplasm. No abnormalities were identified in the regional lymph nodes of the head and neck. The lesion exhibited mild contrast enhancement and expanded extracranially without apparent invasion of the adjacent soft tissues (Figure 1B). Bone-window images revealed thinning of the cortical bone with focal loss of the inner table at the caudal aspect of the calvarium, consistent with an expansile osseous lesion, without a distinct periosteal reaction (Figure 1C). Three-dimensional volume-rendered reconstruction confirmed the exophytic origin of the mass from the left parietal bone (Figure 1D). No evidence of intracranial invasion or regional metastatic disease was identified.

**Figure 2:**
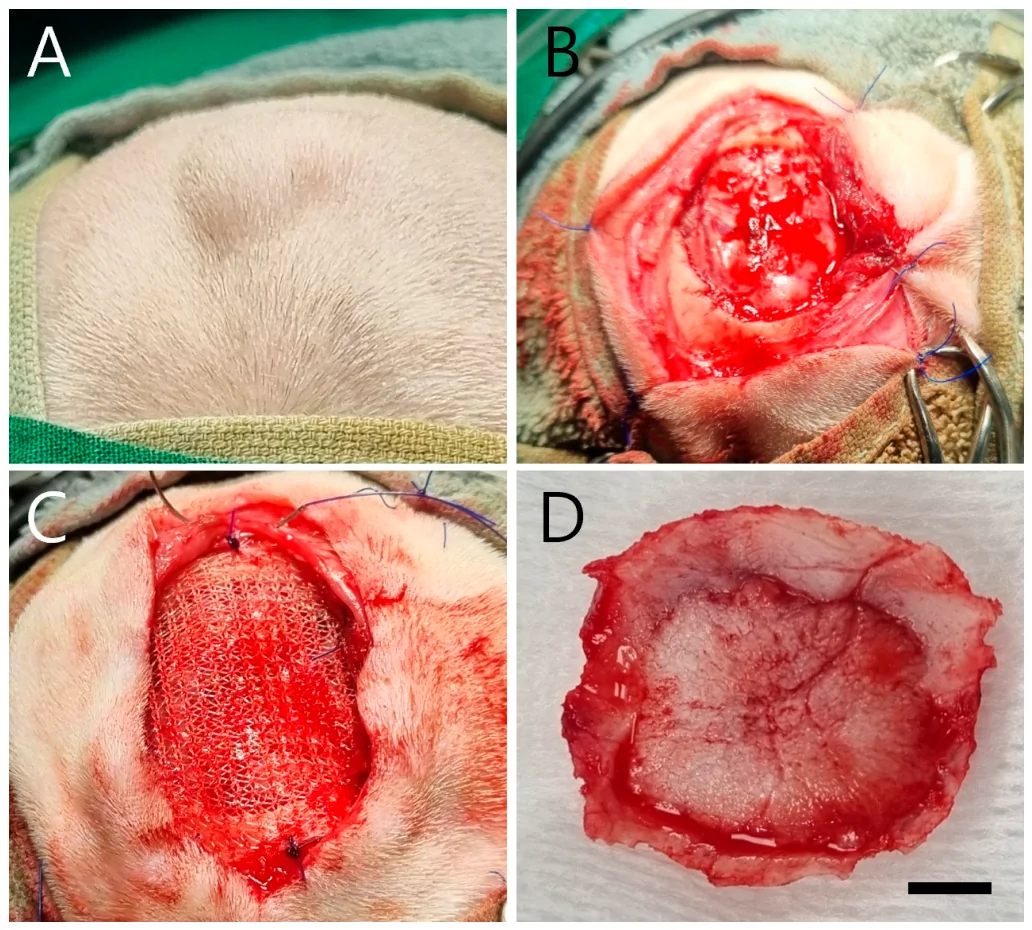
Intraoperative images. (**A**) Preoperative gross appearance of the dome-shaped mass arising from the left parietal bone. (**B**) Calvarial defect following en bloc craniectomy, including removal of the mass and surrounding affected bone. (**C**) Reinforcement of the calvarial defect using polypropylene mesh following en bloc removal of the mass. (**D**) Gross appearance of the excised specimen, consisting of the calvarial mass with the surrounding affected bone removed en bloc (Scale bar = 5 mm).

The dog received intravenous premedication of 0.2 mg/kg butorphanol (Butophan^®^; Myungmoon Pharmaceutical, Seoul, Republic of Korea) and 0.2 mg/kg midazolam (Bukwang Pharmaceutical, Seoul, Republic of Korea). Anesthesia was induced by intravenous injection of 5 mg/kg propofol (Provive Inj.^®^; Myungmoon Pharmaceutical, Seoul, Republic of Korea) and maintained by inhalation of isoflurane (Isoflurane^®^; Choongwae Pharmaceutical, Seoul, Republic of Korea). After preparation of the surgical field (Figure 2A), the skin overlying the mass and the associated temporalis muscle were excised to expose the underlying osseous mass. An en bloc craniectomy including the mass and surrounding affected calvarial bone was performed with care to avoid injury to the dorsal sagittal sinus and bilateral transverse sinuses (Figure 2B). Due to anatomical constraints, a grossly normal bony margin of approximately 3–6 mm was achieved around the lesion. The calvarial defect was reconstructed with a polypropylene mesh to provide structural support and protection of the underlying tissues. (Figure 2C). The surgical site was closed routinely. Recovery from anesthesia was uneventful. The excised mass was submitted for histopathologic evaluation (Figure 2D). The patient was discharged 7 days after surgery. Firocoxib (2.5 mg/kg) was administered orally twice daily for 2 weeks postoperatively. The postoperative course was uneventful. No clinically apparent neurologic deficits, seizures, surgical site infection, wound complications, mesh exposure, or implant-associated complications were observed during the follow-up period.

**Figure 3:**
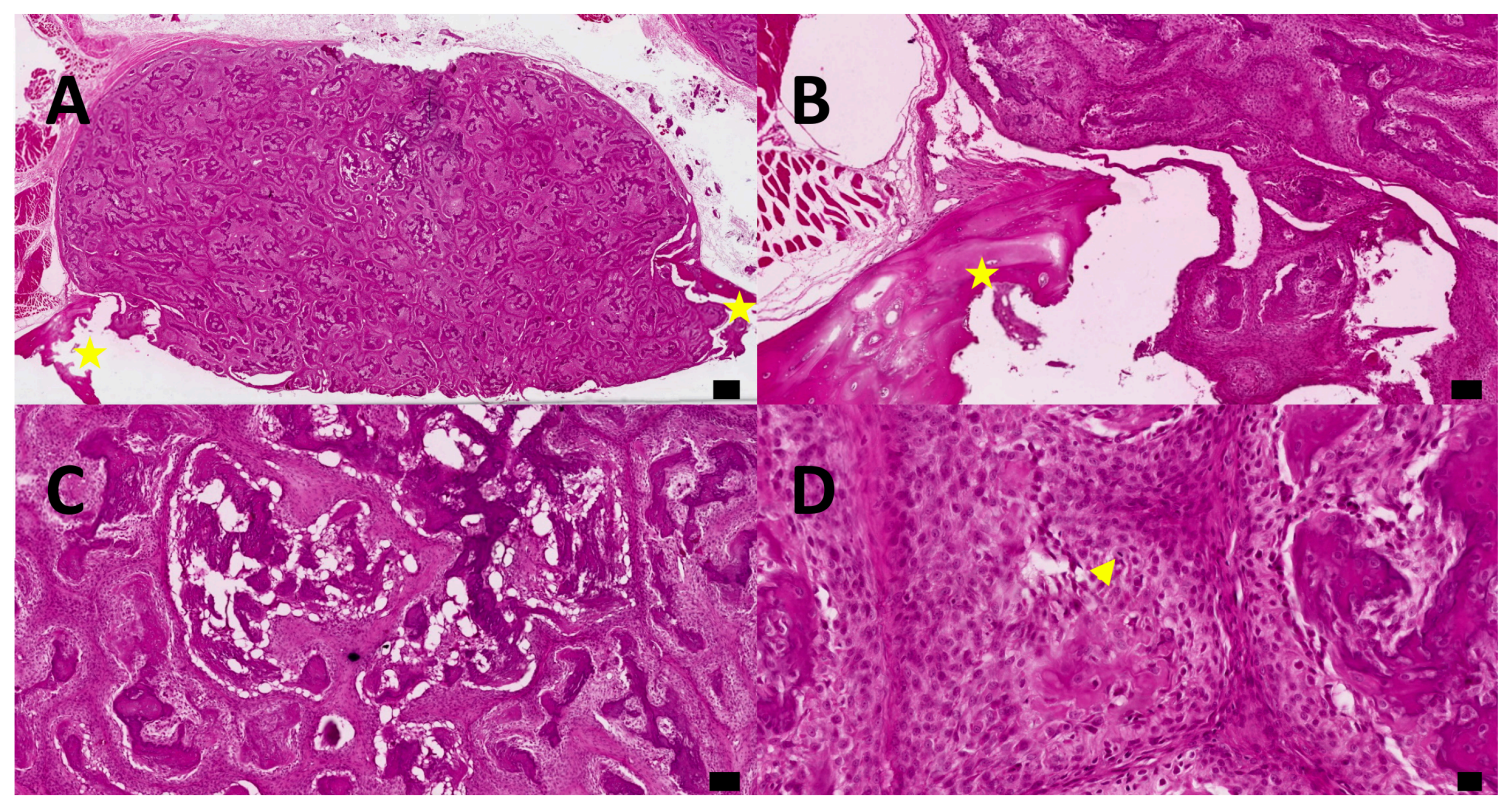
Histopathologic images of the excised mass. (**A**) Low-power view showing a well-demarcated, expansile osseous neoplasm replacing a segment of the calvarial bone, with non-neoplastic mature bone present on both lateral sides (asterisks). (**B**) Neoplasm adjacent to pre-existing mature calvarial bone (asterisk). (**C**) Neoplastic osteoid and woven bone replacing the diploic/medullary compartment, with residual marrow adipocytes entrapped between neoplastic trabeculae. (**D**) High-power view of osteoblastic neoplastic cells showing cellular and nuclear atypia and producing eosinophilic osteoid, with a mitotic figure (arrow). H & E. Scale bars = 500 μm (**A**), 100 μm (**B**,**C**), and 20 μm (**D**).

Histologically, the specimen was a single, firm, ovoid mass arising from the parietal calvarium and measuring 18.3 × 16.6 × 9.4 mm. The tissue was decalcified, routinely processed, and stained with hematoxylin and eosin. The neoplasm was well-demarcated and expansile, replacing a segment of the calvarial bone, with non-neoplastic mature bone present on both lateral sides (Figure 3A). At the tumor margin, neoplastic osteoid and woven bone were contiguous with pre-existing mature calvarial bone (Figure 3B). Within the diploic/medullary compartment, residual marrow adipocytes were entrapped among neoplastic osteoid and woven bone trabeculae (Figure 3C). The neoplasm was composed of round to polygonal, occasionally spindle-shaped osteoblastic cells associated with abundant irregular osteoid. Nuclear pleomorphism was moderate. Neoplastic osteoblasts directly produced eosinophilic osteoid (Figure 3D). Cartilaginous differentiation was not identified. The mitotic count was 3/2.37 mm^2^. Tumor necrosis, vascular invasion, and infiltration into the adjacent extracranial soft tissues were not identified. In the examined sections, non-neoplastic mature bone extended to both lateral osseous resection margins, which were free of neoplastic cells. Overall, the histologic findings supported a central OSA.

Following the diagnosis of OSA, oral imatinib (10 mg/kg) was administered off-label as postoperative adjuvant therapy based on previous reports suggesting that PDGFR-mediated signaling may represent a potential therapeutic target in canine OSA [5]. KIT, PDGFR-α, and PDGFR-β expression was not assessed in the present tumor. During postoperative adjuvant imatinib therapy, no clinically apparent adverse effects requiring dose reduction or discontinuation were observed. Seven months after surgery, follow-up CT and serum biochemical analyses were performed. No evidence of local recurrence, pulmonary metastasis, or distant metastatic disease was identified on CT examination. Serum biochemical findings remained largely unchanged, except that the alkaline phosphatase activity decreased from 157 U/L to 123 U/L (RR, 20–106 U/L) and total cholesterol concentration from 404 mg/dL to 212 mg/dL (RR, 93–340 mg/dL). Three months after the follow-up CT, corresponding to 10 months after surgery, the dog was re-evaluated for continuation of anticancer medication. At that visit, no gross abnormality suggestive of local recurrence was observed on physical examination. However, repeat CT was not performed.

## 3. Discussion

In a retrospective study of 183 dogs with OSA of the maxilla, mandible, or calvarium, the primary tumor site was the maxilla in 80 dogs (43.7%), mandible in 60 dogs (32.8%), and calvarium in 43 dogs (23.5%) [3]. These findings suggest that calvarial involvement was less common than maxillary or mandibular involvement.

In the present case, CT was useful for characterizing the extent of the calvarial lesion, assessing involvement of adjacent structures, and planning surgical excision. Imaging and histopathology are complementary diagnostic modalities for bone tumors, and definitive diagnosis requires histopathologic examination [8]. In this case, definitive diagnosis was established by histopathologic examination, which demonstrated malignant osteoblastic proliferation with osteoid production consistent with OSA [1,8]. Although CT demonstrated an expansile osseous lesion involving the calvarium, imaging alone was insufficient to definitively determine whether the tumor was central or surface-derived. Therefore, the classification as central OSA was based primarily on histopathologic findings. The tumor expanded within and replaced the diploic/medullary compartment. Residual marrow adipocytes were also present within the tumor. Together with the medullary replacement, these findings supported a central origin.

Complete surgical resection with adequate margins is recommended as the primary treatment for OSA of the maxilla, mandible, and calvarium. Tumors arising in the head may present surgical challenges because achieving wide surgical margins is often difficult due to anatomical constraints [3]. In the case reported here, based on the CT findings, en bloc craniectomy and defect reinforcement were performed. Histologically, both examined lateral osseous margins were free of tumor. However, because the entire three-dimensional surgical margin, including the intracranial/dural interface, was not histologically evaluated, an R0 resection could not be confirmed. Polypropylene mesh was selected for reinforcement of the calvarial defect and providing support for overlying soft tissues closure, rather than as a rigid implant. Although rigid materials such as titanium mesh or PMMA may provide greater mechanical protection, non-metallic mesh was chosen in this case to facilitate postoperative CT-based surveillance and to minimize potential interference with future radiation treatment planning. The limited mechanical protection provided by polypropylene mesh compared with rigid reconstructive materials should be considered a limitation of this method. Although polypropylene mesh was selected in part to facilitate potential future radiotherapy, the effect of polypropylene mesh on radiotherapy planning in canine calvarial tumors was not evaluated.

In the study by Selmic et al., dogs treated surgically had a median survival time of 329 days, and histologically tumor-free surgical margins were associated with decreased hazards of progression or recurrence and death [3]. In addition, calvarial tumor location was associated with a greater hazard of local recurrence or progression [3]. These findings suggest that prognosis in calvarial OSA is strongly influenced by local tumor control, which may be particularly difficult to achieve because wide surgical margins are often limited by adjacent critical anatomical structures.

Compared with appendicular OSA, osteosarcoma of the maxilla, mandible, or calvarium appears to have a different pattern of disease progression. In dogs with appendicular OSA, reported median survival times range from 134 to 175 days with surgery alone and from 262 to 413 days with surgery followed by adjuvant chemotherapy [4]. In contrast, dogs with surgically treated OSA of the maxilla, mandible, or calvarium had a median survival time of 329 days, while the reported metastatic rate was lower than that following treatment of appendicular OSA [3]. However, local recurrence or progression occurred more frequently than distant metastasis in dogs with head OSA, and calvarial location was specifically associated with an increased hazard of local recurrence or progression [3]. Thus, compared with appendicular OSA, prognosis in calvarial OSA may be particularly dependent on achieving adequate local tumor control. Several studies have evaluated adjuvant medical therapy for canine OSA [2,3,9]. Kwak et al. reported that a triple-combination regimen consisting of high-dose losartan, toceranib, and carboplatin induced a marked but temporary regression of progressive pulmonary metastatic OSA in a dog, suggesting the potential therapeutic benefit of tumor microenvironment-targeted multimodal therapy [2]. On the other hand, Castro et al. reported a case of canine axial OSA that developed local recurrence despite surgical excision and adjuvant carboplatin chemotherapy, but showed no evidence of metastasis during follow-up [9].

In the present case, imatinib was administered as an adjuvant therapy after surgical resection of OSA. Imatinib was not selected because of proven superiority over conventional adjuvant treatments for canine OSA. Rather, because the role of conventional adjuvant chemotherapy or radiation therapy remains poorly defined for OSA of the maxilla, mandible, and calvarium, imatinib was considered as an off-label adjunctive option based on previously reported biological plausibility regarding PDGFR-mediated signaling in canine OSA. Imatinib, the first tyrosine kinase inhibitor introduced for cancer therapy in the mid-1990s, is an orally administered drug that binds to the ATP-binding pocket of ABL, ARG, PDGFR-α, PDGFR-β, and KIT [6,7]. In veterinary medicine, imatinib has been used to treat various neoplastic diseases, particularly gastrointestinal stromal tumors (GISTs) in dogs, in which activating KIT mutations are frequently identified [10]. Although imatinib has not been approved by the United States Food and Drug Administration for veterinary use, it has been widely employed off-label in dogs with favorable clinical outcomes not only for GIST but also other neoplasms, such as mast cell tumors [11,12,13].

Although clinical evidence supporting imatinib therapy for canine OSA is limited, previous studies have demonstrated PDGFR-α and PDGFR-β expression in canine OSA tissues, suggesting that PDGFR-mediated signaling may represent a potential therapeutic target in this tumor type [5]. Therefore, imatinib was considered an off-label therapeutic option in this case. However, KIT, PDGFR-α, and PDGFR-β expression was not evaluated in the present tumor. Thus, imatinib was selected based on previously reported biological plausibility rather than molecular confirmation of target expression or activation in this case. Consequently, the independent therapeutic contribution of imatinib cannot be determined from this single case. No standardized dosage or treatment duration has been established for imatinib therapy in canine OSA. Therefore, the dose of 10 mg/kg and the planned 1-year treatment duration used in this case were empirically selected based on previous off-label use of imatinib in canine neoplasms, expected tolerability, and owner consent. The optimal dose, duration, safety, and efficacy of imatinib in canine OSA remain undetermined.

In the present case, despite the high metastatic potential of canine OSA, no evidence of local recurrence, pulmonary metastasis, or distant metastatic disease was detected on follow-up CT performed seven months after surgery. In appendicular canine OSA, wide resection generally requires bone margins of at least 3 cm and 2–3 cm margins in the surrounding muscle and other soft tissues [14]. However, achieving such margins is often impossible in calvarial OSA because of anatomical constraints. Despite performing only a marginal-to-wide resection dictated by these anatomical limitations in the present case, no evidence of local recurrence or distant metastasis was observed on CT at seven months after surgery, and no gross abnormality suggestive of local recurrence was noted on physical examination at 10 months after surgery. However, this follow-up duration should be interpreted as short- to intermediate-term surveillance rather than evidence of durable long-term tumor control. Because local recurrence or progression may occur later in dogs with OSA of the maxilla, mandible, or calvarium, longer follow-up with serial imaging and clinical outcome assessment is required to evaluate long-term local control and metastatic behavior. Although imatinib was administered as postoperative adjunctive therapy, its therapeutic contribution cannot be determined in this case. KIT, PDGFR-α, and PDGFR-β expression was not assessed in the tumor, and imatinib was therefore selected based on previously reported biological plausibility rather than molecular confirmation in this patient. In addition, because surgical excision was performed, and adjunctive imatinib was administered concurrently, the absence of recurrence or metastasis during the available follow-up period cannot be attributed specifically to imatinib or distinguished from the effects of surgery, margin status, or the natural course of the disease. Nevertheless, further studies involving larger numbers of cases with molecular target assessment are required to determine whether imatinib has therapeutic efficacy in canine OSA.

This case report has several limitations. First, because this was a single clinical case, the findings cannot be generalized to all dogs with calvarial OSA. Second, although follow-up whole-body CT performed seven months after surgery showed no evidence of local recurrence or distant metastasis, and clinical re-evaluation at 10 months after surgery revealed no gross abnormality suggestive of local recurrence, the follow-up period was limited. Therefore, long-term outcome, survival time, delayed local recurrence, and metastatic progression could not be fully assessed. In addition, because this was a single clinical case treated with surgical excision and concurrent postoperative adjunctive therapy, the individual contributions of surgery, margin status, imatinib, and the natural course of the disease could not be determined. Another limitation is that adverse events and treatment tolerability were not evaluated using a standardized toxicity grading system. Therefore, the absence of clinically apparent complications in this case should not be interpreted as evidence of the general safety of polypropylene mesh reconstruction or postoperative adjuvant therapy. Finally, although both examined lateral osseous margins were free of tumor, the entire three-dimensional surgical margin, including the intracranial/dural interface, could not be evaluated histologically. Therefore, an R0 resection could not be confirmed. Further long-term follow-up and accumulation of additional cases are needed to better characterize the prognosis and optimal treatment strategy for canine calvarial OSA.

## 4. Conclusions

This case describes the clinical course of a dog with calvarial OSA treated with en bloc craniectomy, calvarial defect reinforcement, and postoperative adjunctive imatinib therapy. No evidence of local recurrence or metastatic disease was identified during the 7-month follow-up period. However, because KIT, PDGFR-α, and PDGFR-β expression was not assessed and this was a single clinical case, the independent therapeutic contribution of imatinib could not be determined. This report provides additional clinical information regarding postoperative CT monitoring and adjunctive imatinib administration in canine calvarial OSA. Further studies involving larger case series, longer follow-up periods, and molecular target assessment are warranted.

## Data Availability

The data presented in this study are available on request from the corresponding author to protect the privacy of client-owned animals and their owners’ medical records.

## Abbreviations

The following abbreviations are used in this manuscript:

OSA	Osteosarcoma
CT	Computed tomography
RR	Reference range
GIST	Gastrointestinal stromal tumor
